# Revolutionizing anti-cancer drug discovery against breast cancer and lung cancer by modification of natural genistein: an advanced computational and drug design approach

**DOI:** 10.3389/fonc.2023.1228865

**Published:** 2023-09-25

**Authors:** Shopnil Akash, Shabana Bibi, Partha Biswas, Nobendu Mukerjee, Dhrubo Ahmed Khan, Md. Nazmul Hasan, Nazneen Ahmeda Sultana, Md. Eram Hosen, Yousef A. Bin Jardan, Hiba-Allah Nafidi, Mohammed Bourhia

**Affiliations:** ^1^ Faculty of Allied Health Science, Department of Pharmacy, Daffodil International University, Dhaka, Bangladesh; ^2^ Department of Biosciences, Shifa Tameer-e-Millat University, Islamabad, Pakistan; ^3^ Laboratory of Pharmaceutical Biotechnology and Bioinformatics, Department of Genetic Engineering and Biotechnology, Jashore University of Science and Technology, Jashore, Bangladesh; ^4^ Department of Microbiology, West Bengal State University, Kolkata, India; ^5^ Professor Joarder DNA and Chromosome Research Laboratory, Department of Genetic Engineering and Biotechnology, University of Rajshahi, Rajshahi, Bangladesh; ^6^ Department of Food Science, Faculty of Agricultural and Food Sciences, Laval University, Quebec City, QC, Canada; ^7^ Department of Pharmaceutics, College of Pharmacy, King Saud University, Riyadh, Saudi Arabia; ^8^ Laboratory of Chemistry and Biochemistry, Faculty of Medicine and Pharmacy, Ibn Zohr University, Laayoune, Morocco

**Keywords:** drug design, genistein, breast cancer, lung cancer, *Glycyrrhiza glabra*, molecular docking, molecular dynamics simulation

## Abstract

Breast and lung cancer are two of the most lethal forms of cancer, responsible for a disproportionately high number of deaths worldwide. Both doctors and cancer patients express alarm about the rising incidence of the disease globally. Although targeted treatment has achieved enormous advancements, it is not without its drawbacks. Numerous medicines and chemotherapeutic drugs have been authorized by the FDA; nevertheless, they can be quite costly and often fall short of completely curing the condition. Therefore, this investigation has been conducted to identify a potential medication against breast and lung cancer through structural modification of genistein. Genistein is the active compound in *Glycyrrhiza glabra* (licorice), and it exhibits solid anticancer efficiency against various cancers, including breast cancer, lung cancer, and brain cancer. Hence, the design of its analogs with the interchange of five functional groups—COOH, NH_2_ and OCH_3_, Benzene, and NH-CH_2_-CH_2_-OH—have been employed to enhance affinities compared to primary genistein. Additionally, advanced computational studies such as PASS prediction, molecular docking, ADMET, and molecular dynamics simulation were conducted. Firstly, the PASS prediction spectrum was analyzed, revealing that the designed genistein analogs exhibit improved antineoplastic activity. In the prediction data, breast and lung cancer were selected as primary targets. Subsequently, other computational investigations were gradually conducted. The mentioned compounds have shown acceptable results for *in silico* ADME, AMES toxicity, and hepatotoxicity estimations, which are fundamental for their oral medication. It is noteworthy that the initial binding affinity was only −8.7 kcal/mol against the breast cancer targeted protein (PDB ID: 3HB5). However, after the modification of the functional group, when calculating the binding affinities, it becomes apparent that the binding affinities increase gradually, reaching a maximum of −11.0 and −10.0 kcal/mol. Similarly, the initial binding affinity was only −8.0 kcal/mol against lung cancer (PDB ID: 2P85), but after the addition of binding affinity, it reached −9.5 kcal/mol. Finally, a molecular dynamics simulation was conducted to study the molecular models over 100 ns and examine the stability of the docked complexes. The results indicate that the selected complexes remain highly stable throughout the 100-ns molecular dynamics simulation runs, displaying strong correlations with the binding of targeted ligands within the active site of the selected protein. It is important to further investigate and proceed to clinical or wet lab experiments to determine the practical value of the proposed compounds.

## Graphical Abstract

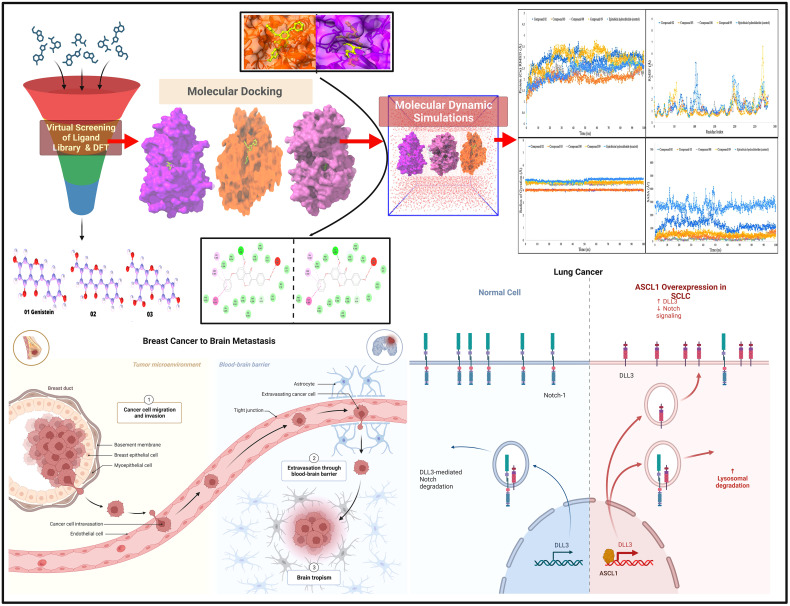

## 1 Introduction

The human body is composed of countless tiny cells, and after a specific period, all the cells die and are replaced by new ones through a continuous process. When these cells develop out of control, they are known as tumors (1). Tumors can be classified into two types: benign and malignant. In detail, the uncontrolled growth of tissue is referred to as neoplasia or a tumor. Benign tumors are generally a result of regular cell function; furthermore, they do not become cancerous unless they spread to other parts of the body. When neoplasia extends beyond the vicinity of normal cells, it is termed a malignant tumor or cancer. In this case, cells continuously undergo uncontrolled differentiation, giving rise to what are known as cancer cells (2–4). Cancers initially grow similarly to benign tumors, and subsequently, the cells of the benign tumor may transform into malignant cells, giving rise to cancer. However, it is not guaranteed that all benign tumors will progress to cancer. Metastasis is a stage of cancer where malignant cells invade other areas and spread to neighboring tissues through the bloodstream and lymphatic system (5–8).

Consequently, cancer cells develop and grow uncontrolled, leading to a patient**’**s death. Based on current estimates, metastasis is accountable for approximately 90% of cancer fatalities (9, 10). Breast cancer is the most prevalent malignant growth in women, representing a complex and life-threatening disorder, particularly for female patients. Furthermore, breast cancer is among the most feared and widespread forms of cancer in women. It arises when a woman**’**s breast cells undergo irregular growth, especially within the milk-producing glands of the body (11, 12). It is estimated that approximately 50% of breast cancers occur in women with no known risk factors for breast cancer (over 40 years) (13, 14). According to many research findings, sexual dysfunction contributes significantly to breast cancer. Eventually, this is linked to various physiological factors, such as anxiety over fertility (15, 16).

Lung cancer is caused by a variety of factors, with the most common being smoking. Some lung cancer cases among individuals with no smoking history may be attributed to exposure to radon (222Rn), which emanates from building materials. Clinically, only a small percentage of NSCLC (non-small cell lung cancer) patients are diagnosed at an early stage, while the majority present with advanced or even metastatic disease, resulting in a 5-year survival rate of less than 15%. Despite some therapeutic advancements in conventional treatments over the last several decades, the prognosis for lung cancer remains challenging due to its unique physiological characteristics and the frequent occurrence of tumor escape (17, 18). Nowadays, lung cancer is regarded as one of the deadliest oncological diseases globally. Literature reports that more than 80% to 85% of all lung cancers are categorized as NSCLC. In 2020, lung cancer accounted for more than 2.2 million new cases and approximately 1.8 million deaths (19, 20).

Currently, numerous chemotherapeutic agents are available in the market, which have dangerous side effects such as damage to lung tissue, heart problems, infertility, kidney problems, and nerve damage (peripheral neuropathy) (21). Evidence illustrates that effective and innovative medication is very much needed to treat this disease with lower side effects. Developing an effective and commercially available drug is time-consuming and requires a lot of human resources, time, and money.

Therefore, we attempted to identify an active compound within natural plant sources and subsequently modified it to enhance its efficiency compared to the original compound. Our focus was on *G. glabra* (licorice) as a potential source for identifying such a compound. *G. glabra* was a common ingredient in numerous Ancient Greek medicinal formulations, and it contains a variety of bioactive compounds, including genistein, daidzin, ononin, and formononetin (22). Among them, it has been revealed that genistein can prevent the development of different cancer cells. The interruption of the cell cycle, which brings about a stoppage to the proliferation of cells, could be the causative agent of the growth inhibition observed in cancer cells (23). Moreover, genistein has strong anticancer efficiency against numerous diseases such as breast cancer, lung cancer, and brain cancer. Thus, although licorice is composed of numerous bioactive compounds, we have chosen genistein as our targeted drug (24). Hence, to save time, money, and resources, the computational technique should be a helpful method, and this is why this investigation, via structural modification of genistein (C_15_H_10_O_5_) belonging to a group of chemicals classified as **“**isoflavones**”**, has been performed (25).

Breast cancer and lung cancer need to be well treated due to the lack of potential medicine. In this study, we investigated the natural compound genistein and its derivatives for breast and lung cancer. Computer-aided drug design (CADD) techniques were applied to the dataset, such as PASS predictions, protein–ligand interactions, molecular dynamics (MD) simulation analysis, and ADMET profile estimations. Therefore, our results will add knowledge to breast and lung cancer drug design and development.

## 2 Compound selection criteria

Genistein (C_15_H_10_O_5_) is classified as an isoflavone, which places it among the flavonoid family of compounds. It is a phytoestrogen that comes predominantly from legumes as its source material. It is a naturally occurring chemical component. It is said that it may improve several aspects of one**’**s health, including protection against osteoporosis, a decrease in the likelihood of developing cardiovascular disease, relief from the symptoms of post-menopause, and anticancer capabilities (26). In the past, a wide variety of research projects, both *in vitro* and *in vivo*, have been carried out to examine the possible anti-inflammatory effects of genistein (27, 28). Hence, several studies have shown that exposure to genistein may cause cancer cells to commit suicide via a variety of pathways, including those involved in cell signaling. Evidence of the apoptotic nature of genistein on breast cancer cells is emphasized, demonstrating that genistein may play a promising function. These data come from both *in vitro* and *in vivo* studies (29).

### 2.1 Genistein function in early stages of cancer development

Several studies have shown that early introduction to genistein might lower the risk of developing breast cancer (30). Mammary terminal end buds are ducts that may be detected in young animals. These ducts include a significant number of undifferentiated cells that are susceptible to damage from carcinogens. The administration of genistein to young rats resulted in a reduction in the number of terminal end buds and an expansion of the number of lobules. Scientists concluded that pre-pubertal and adult treatment of experimentally induced breast cancer in genistein-protected rats should take place between the time of birth and the pre-pubertal stage of mammary gland growth for there to be a beneficial factor of genistein (29, 31). Scientists concluded that genistein acts as a chemo-preventive medicine during the pre-pubertal phase, which they feel correlates to the adolescent phase in human life. As a result of this research, it has been shown that one of the cellular mechanisms of action of genistein is a rise in the proliferation and differentiation of the breast (29).

## 3 Materials and methods

### 3.1 PASS prediction

We employed the PASS online web tool (http://way2drug.com/PassOnline/predict.phpp) to assess the antibacterial, antifungal, antiviral, and antineoplastic activity PASS prediction spectrum of the reported drug molecules. To begin, the structures of the drug molecules were drawn using ChemBioDraw and then converted into their SMILES formats with the assistance of the SwissADME free online tool (http://www.swissadme.ch).

Subsequently, the SMILES forms were uploaded to the PASS website to determine the antifungal, antiviral, and antineoplastic activity PASS prediction spectrum. This web tool incorporates more than 4,000 types of features, encompassing both drug and non-drug activities, enabling the identification of potential bioactive compounds with 90% authenticity (32, 33).

The measurement values provided by PASS are described by P**_a_** (probability for active molecule) and P**_i_** (probability for inactive molecule). For a molecule to be considered potential, the P_a_ and P_i_ values should fall within the range of 0.00 to 1.00, with P_a_ + P_i_ ≠ 1. In this context, biological actions with P_a_ > P_i_ are deemed probable for the selected drug molecule. PASS measurement values are described by P_a_ (probability for active molecule) and P_i_ (probability for inactive molecule). To be a potential molecule, the P_a_ and P_i_ values should range from 0.00 to 1.00, and generally, P_a_ + P_i_ ≠ 1. The biological actions with P_a_ > P_i_ are thought probable for a selected drug molecule (34, 35).

### 3.2 Preparation of a ligand dataset

**Figure 1:**
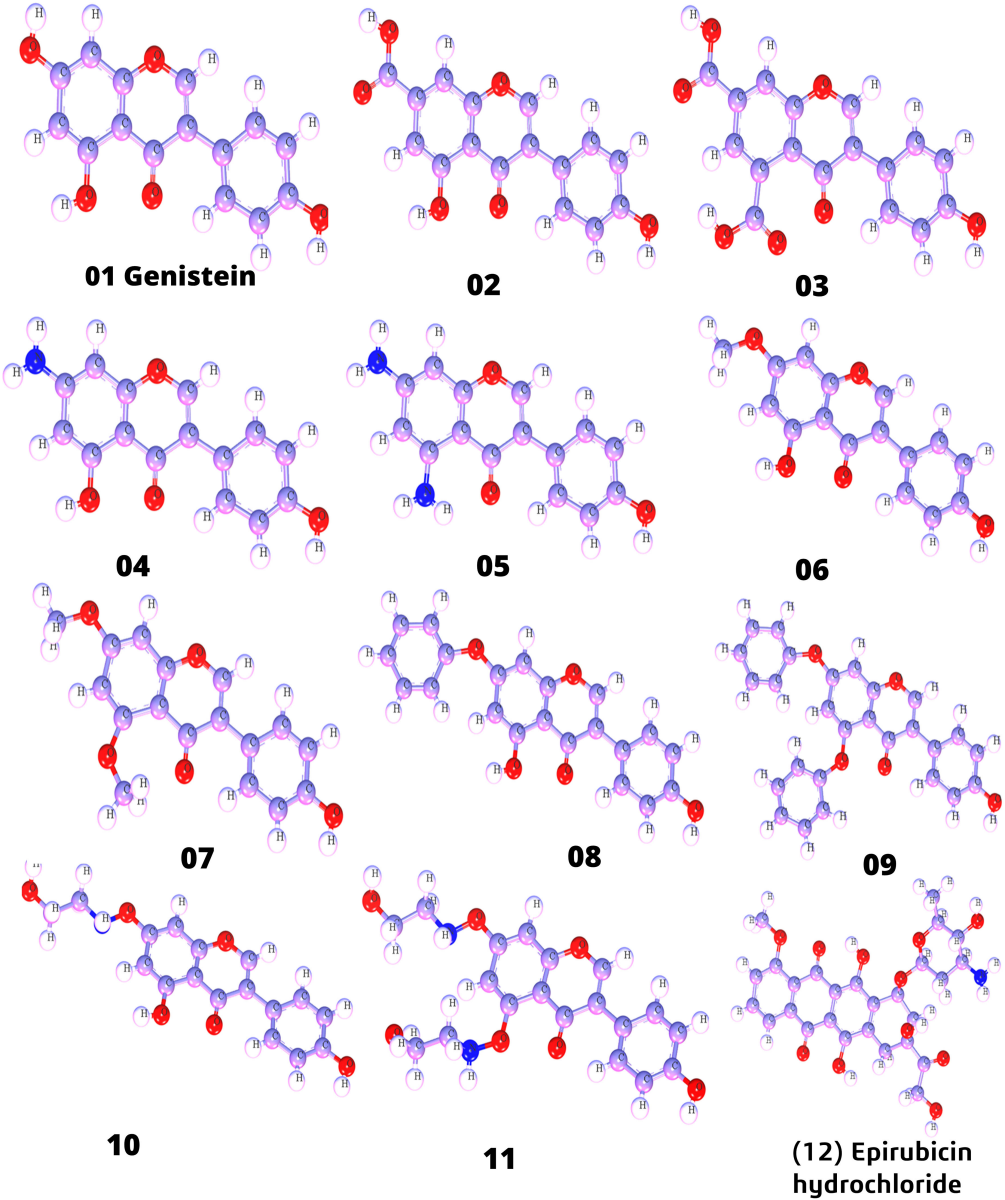
Three-dimensional molecular structure (designed analogs).

Firstly, genistein was prepared and drawn with ChemBioDraw (32). In this case, five activated functional groups have been chosen, namely, —COOH, NH_2_, OCH_3_, Benzene, and NH-CH_2_-CH_2_-OH. The parent molecule was genistein, which consists of a hydroxyl group in different positions of the aromatic ring. Thus, while designing analogs of genistein, the hydroxyl group of genistein was modified, and substitution of —COOH, NH_2_, OCH_3_, Benzene, and NH-CH_2_-CH_2_-OH functional groups. After that, these modified structures of genistein were optimized with the help of material studio software (36) and saved as a PDB file type for further computational studies such as molecular docking, ADMET, protein–ligand interaction, MD simulation, and Lipinski rule. In **Figure 1**, optimized structures are displayed.

### 3.3 Determination of the ADMET profile

To determine the pharmacokinetics and toxicity of the drugs as mentioned above, we used the web-based servers pkCSM (http://biosig.unimelb.edu.au/pkcsm/prediction) (37). This online database has been used to assess the pharmacokinetics parameters related to drug absorption, metabolism, and toxicity features. This machine learning method predicts the pharmacokinetics parameter based on the structural similarity of the chemical associated with known ADMET features. Moreover, this is the most trusted and well-known machine learning-based tool for forecasting ADMET (38). The essential data include water solubility Log S, human intestinal absorption, Caco-2 permeability, VDss (human), blood–brain barrier (BBB) permeability, CYP450 1A2 inhibitor, CYP450 2C9 substrate, total clearance (ml/min/kg), and renal OCT2 substrate. Then, toxicity data including AMES toxicity, max. tolerated dose, oral rat acute toxicity (LD_50_), oral rat chronic toxicity, hepatotoxicity, skin sensitization, and *Tetrahymena pyriformis* toxicity were analyzed. Lipinski**’**s rule of five is often used to assess the drug-likeness of chemical or bioactive molecules. Any chemical compound should be an oral drug if it follows Lipinski**’**s rule of five, which includes molecular mass and the number of hydrogen bond acceptors, the number of hydrogen bond donors, the number of rotatable bonds, molar refractivity, and bioavailability score. Lipinski**’**s rule of five, topological polar surface area (TPSA), and solubility were estimated using this method. Additionally, to determine this feature, the SwissADME (http://www.swissadme.ch/index.php) online application was used, and the required data were listed (39).

### 3.4 Protein preparation and molecular docking

**Figure 2:**
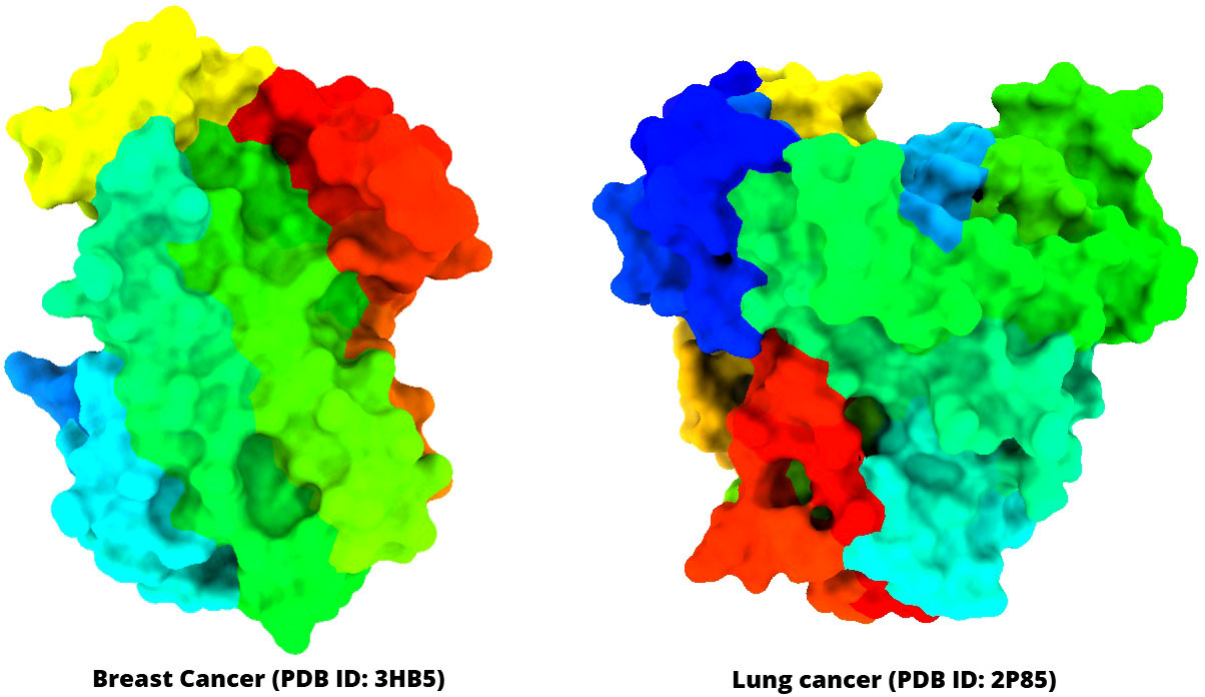
Three-dimensional structure of selected breast cancer and lung cancer target proteins used in this study.

The three-dimensional (3D) protein of breast cancer (PDB ID: 3HV5) and lung cancer (PDB ID: 2P85) was selected and downloaded from the Protein Data Bank (PDB) as a PDB file type (**Table 1**) (40, 41). Gradually, all hetero atoms, excess water molecules, and other impurities were cleaned away by implementing Pymol application (42). Finally, the optimized selected compounds were picked up for molecular docking study with breast cancer (PDB ID: 3HV5) and lung cancer (PDB ID: 2P85). In the end, the PyRx software was used to create the molecular docking interaction with AutoDock Vina (43). The protein information and tertiary structure are displayed in **Figure 2**.

**Table 1:** Summary of ligand results calculated by the PASS prediction tool.

Ligand no.	Antiviral		Antibacterial		Antifungal		Antineoplastic	
	P_a_	P_i_	P_a_	P_i_	P_a_	P_i_	P_a_	P_i_
01	0.190	0.106	0.394	0.031	0.502	0.030	0.750	0.018
02	0.190	0.105	0.442	0.023	0.264	0.037	0.505	0.070
03	0.160	0.148	0.407	0.028	0.399	0.050	0.343	0.128
04	0.188	0.108	0.401	0.030	0.414	0.047	0.651	0.035
05	0.507	0.048	0.406	0.028	0.282	0.089	0.771	0.016
06	0.096	0.048	0.377	0.036	0.489	0.032	0.735	0.020
07	0.299	0.036	0.340	0.046	0.444	0.041	0.712	0.024
08	0.168	0.137	0.349	0.044	0.473	0.035	0.776	0.025
09	0.184	0.114	0.312	0.056	0.427	0.044	0.696	0.027
10	0.339	0.067	0.331	0.049	0.307	0.078	0.742	0.019
11	0.294	0.094	0.265	0.075	0.241	0.111	0.783	0.014

### 3.5 Molecular dynamics simulation

The MD simulations of the targeted breast cancer protein with the selected ligand complex have been conducted to establish the binding accuracy of the five newly developed drug candidates (L02, L03, L08, L09, and epirubicin hydrochloride) to the targeted protein breast cancer (PDB ID 3HB5). The **“**Desmond v3.6 program**”** in Schrödinger (https://www.schrodinger.com/) has been implemented for MD simulation of the macromolecular protein and ligand complex in a high configuration desktop of Linux mode to determine the thermodynamic stability (44). For this methodology, a pre-determined TIP3P water technique was introduced to ensure a certain capacity with the rhombohedral periodic bounding box geometry at a value of 10. This strategy was designed to keep the volume constant. Suitable ions, such as 0+ and 0.15 M salt, were selected to neutralize the foundation electronically, and these ions have been arbitrarily distributed throughout the suitable solvent. After developing the solvency protein system with a ligand complex, the system framework was decreased and loosened via the predefined protocol created by merely making use of the Desmond module**’**s OPLS3e parameters for the force field and the implementation details (45). There were 50 PS capture intervals of 1.2 kcal/mol each in NPT components using the temperature amalgamation of Nose-Hoover and the isotropic method, which were held at 300 K and one atmospheric pressure.

#### 3.5.1 Simulation trajectory analysis

The Schrodinger Maestro program version 9.5 was used to create every MD simulation illustration. The macromolecular Interaction Schematic of the Desmond mode in the Schrodinger application was utilized to examine the simulation activity; correspondingly, this analysis was performed so that the accuracy of the MD could be verified. RMSD, RMSF, intramolecular hydrogen bonds, protein–ligand contacts (P-L), solvent-accessible surface area (SASA), the radius of gyration (Rg) score, MolSA, and polar surface area (PSA) were used to evaluate the stability of the protein–ligand complex structure.

#### 3.5.2 Root mean square deviation analysis

Root mean square deviation (RMSD) is a measure utilized in MD simulations that measures how far a single atom has moved over a certain amount of time compared to a reference point (46). First, the RMSD of macromolecular structures such as side chains, backbone, and heavier particles is computed. Next, the RMSD of protein fit ligand atoms from all time frames is generated, and then they are adjusted and evaluated in comparison to the required time (100 ns). The following equation may be used to calculate the RMSD of an MD simulation with period x (Eq. 1).


(1) RMSDx=(1/N)∑(i=1)^N▒〚(r'i)〛(tx))−ri(tref))2

Here, N shows the number of atoms chosen; tref is the reference time, and r**’** conveys the placement of the bit selected in the system x after superimposing the point of the reference system.

#### 3.5.3 Root mean square fluctuation analysis

The root mean square fluctuation (RMSF) has been mainly used to identify and monitor variations in dynamic structure within the binding protein (47). The RMSF value of an MD simulation of a protein with a particular number of residues may be determined with the help of the following equation (Eq. 2), which can be found in the previous sentence (Eq. 1).


(2) RMSFi=(1/T)∑(t=1)^T▒〚<(r'i〛(t))−ri(tref))2>

Here, T generally denotes the trajectory time; t_ref_ denotes the given time; r**’** denotes the location of the reported molecules in a framework I after transposing on the given frame, and (<>) suggests the average square distances covered over residues.

## 4 Results and analysis

### 4.1 PASS prediction

The PASS prediction parameter is used to assess the probability to be active (Pa) and Pi probability to be inactive in the potential bioactive compounds (48). The values of P_a_ and P_i_ range might be 0.00 to 1.0, and importantly, P_a_ + P_i_ ≠ 1, indicating that a compound cannot be fully active and inactive simultaneously (49). In the above discussion, our reported chemical compounds (**Table 1**) have carried the most potent antineoplastic activity, which is above P_a_ value 0.650+ by most of the molecules and owing to Ligand no 01, 05, 06, 07, 08, 10 and 11 showed the highest Pa value (Pa > 0.750, Pa > 0.771, Pa>735, Pa>0.712, Pa> 0.776, Pa> 0.742, and Pa > 0.783), where the antiviral, antibacterial, and antifungal values are reported as 0.096–0.339, 0.265–0.442, and 0.241–0.502. Such high P_a_ values underscore their potential as effective antineoplastic agents. The primary concern is to determine the antineoplastic properties. We also calculated the antiviral, antibacterial, and antifungal features to assess the potential P_a_ and P_i_ parameters and compare them with the antineoplastic activity. It became evident that the probability of being active (P_a_) and inactive (P_i_) is more favorable for antineoplastic activity compared to antiviral, antibacterial, and antifungal activities. Hence, based on these features, breast cancer and lung cancer target proteins have been chosen from the PDB for further investigation.

### 4.2 Lipinski rule, pharmacokinetics, and drug-likeness

The drug (ADMET) profiles of biologically active compounds, such as membrane permeability, gastrointestinal absorption and bioavailability, partition coefficient (log P), molecular weight (MW), and the number of hydrogen bond acceptors/donors, represent overall features of chemical acceptances and drug-likeness in accordance with the Lipinski rule, among other things (50). It has been determined that all the newly developed molecules completely satisfied all the criteria and follow the Lipinski rule after studying the Lipinski rule, pharmacokinetics, and drug-likeness (**Table 2**). Whereas, the features of the G.I. absorption rate are very high according to the obtained result, and only drug molecule 09 has a lower G.I. absorption rate. Finally, it has been reported that all the mentioned ligands have better oral bioavailability scores according to the finding from the SwissADME (http://www.swissadme.ch/index.php) online application. Consequently, it could be summarized that these mentioned drugs should be used as an oral medication against life-threatening breast cancer.

**Table 2:** Summary of ligand calculated results for Lipinski rule, pharmacokinetics, and drug-likeness activities.

Ligand no.	NBR*	HBA*	HBD*	TPSA* (Å^2)^	Lipinski rule	MW*	BS*	GIA*
01	01	05	03	90.90	Yes	270.24	0.55	High
02	02	06	03	107.97	Yes	298.25	0.56	High
03	02	07	03	125.02	Yes	326.26	0.56	High
04	01	04	03	96.69	Yes	269.25	0.55	High
05	01	03	03	102.48	Yes	268.27	0.55	High
06	02	05	02	79.90	Yes	284.26	0.55	High
07	03	05	01	68.90	Yes	298.29	0.55	High
08	03	05	02	79.90	Yes	346.33	0.55	High
09	05	05	01	68.90	Yes	422.33	0.55	Low
10	05	07	04	112.16	Yes	329.30	0.55	High
11	09	09	05	133.42	Yes	388.37	0.55	High

TPSA, Topological polar surface area; NBR, Number of rotatable bonds; HBA, Hydrogen bond acceptor; HBD, Hydrogen bond donor; M. W, Molecular weight; G. I. A, Gastrointestinal absorption.

### 4.3 Molecular docking analysis against breast and lung cancer targeted proteins

Structure-based drug design, such as the molecular docking technique, plays a fundamental role. This technique**’**s principal purpose is to find probable binding geometries of a suspected receptor of a specified 3D structure with a biological target using molecular docking simulations (51). Using the PyRx AutoDock Vina tool, probable binding affinities and interaction sites with the active site of breast and lung cancer-targeted proteins have been determined (52). The potential binding energies of the targeted site of the breast and lung cancer targeted proteins are summarized in **Table 3** for all the reported compounds. The standard docking score has been regarded as −6.0 kcal/mol to be active molecules (53, 54). The range of docking score is −8.4 kcal/mol to −11.00 kcal/mol for breast cancer (PDB ID: 3HB5), while the binding affinity range for lung cancer is −7.2 kcal/mol to −9.5 kcal/mol. According to the finding obtained from docking analysis, the maximum score has been reported as −11.0 kcal/mol and −10.0 kcal/mol in ligand nos. 08 and 09 against PDB ID: 3HB5, while the maximum docking score of −9.5 kcal/mol was obtained against lung cancer (PDB ID: 2P85). Finally, it could be summarized that the designing derivatives of genistein (C1_5_H_10_O_5_) are much better compared with the FDA-approved epirubicin hydrochloride. Thus, they should be suggested as treatment against both breast cancer and lung cancer and might be potential drug candidates.

**Table 3:** Binding affinities of docked ligand calculated against breast and lung cancer targeted proteins.

Ligand no.	Breast cancer (PDB ID: 3HB5)	Lung cancer (PDB ID: 2P85)
	kcal/mol	kcal/mol
01	−8.7	−8.0
02	−9.0	−7.4
03	−9.1	−8.8
04	−8.6	−8.3
05	−8.8	−7.9
06	−8.5	−7.5
07	−8.4	−7.2
08	−11.0	−8.7
09	−10.0	−9.5
10	−8.9	−7.4
11	−8.4	−8.2
Epirubicin hydrochloride	−8.2	−7.7

### 4.4 Molecular docking and interaction analysis

**Figure 3:**
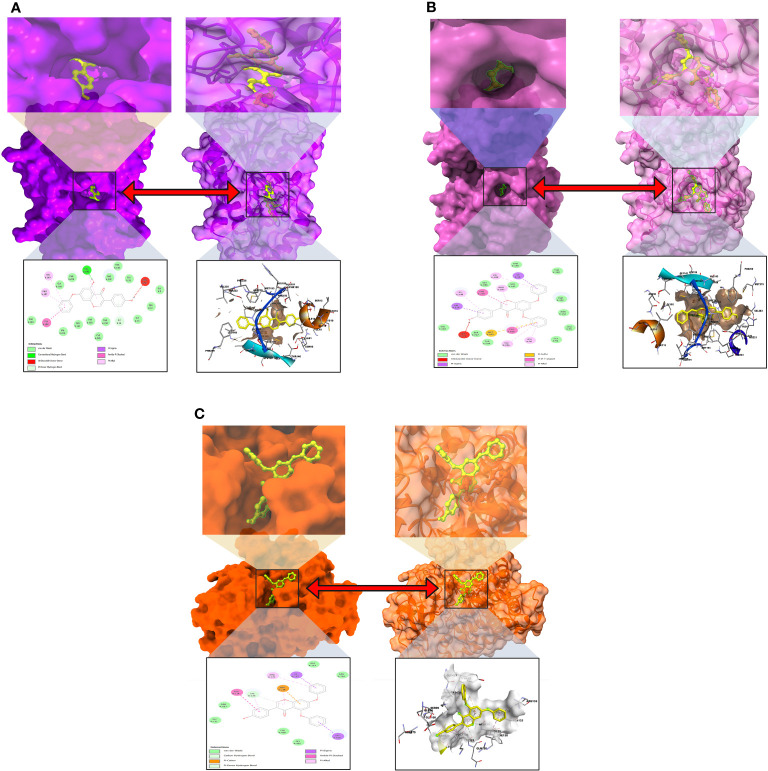
Molecular docking pocket and active site analysis. **(A)** Breast cancer complex with ligand 08. **(B)** Breast cancer complex with ligand 09. **(C)** Lung cancer complex with ligand 09.

The interactions between the inhibitor and the targeted protein active side, docking pocket ligand–protein, are graphically represented in **Figure 3**. In the three-dimensional configuration, the active side of the amino acid residue has been analyzed. In most cases, the number of hydrophobic bond residues is higher than the others, such as hydrogen as electrostatic, van der Waals interactions, and halogen bonds. This protein–ligand interaction has been designed by importing the protein–ligand complex file into the Discovery Studio Visualizer and UCSF Chimera (53). The reported active sites and binding pocket of breast cancer complex with ligand 08 and ligand 09 and lung cancer complex with ligand 09 are displayed in **Figures 3A–C**.

### 4.5 ADMET profile investigation

The pkCSM methodology approach was applied to investigate the pharmacokinetic and ADMET physicochemical features of the reported drugs, such as water solubility Log S, Caco-2 permeability, VDss (human) BBB permeability, CYP450 1A2 inhibitor, CYP450 2C9 inhibitor, total clearance, and renal OCT2 substrate, while all these features influence the absorption, distribution, metabolism, and excretion of drug molecules. This feature is essential for a drug to be physiologically active and reach its target organ in the physiological system by a sample concentration (55).

The investigation has shown that each compound’s water solubility range is different. The solubility range is −2.892 to −5.035, which indicates that most of the compounds are highly soluble in physiological conditions; in comparison, only ligand 08 is poorly soluble. Noted that the solubility feature of the compounds is categorized as insoluble if the value is more negative than −10. The solubility ranges from poorly soluble to highly soluble, with values ranging from −10 to greater than zero, respectively. Compounds with values between −10 and −6 are considered poorly soluble, while those higher than −6 and less than −4 are classified as moderately soluble; soluble compounds might be between −4 to −2, and when the value is between −2 and 0, compounds are very soluble (56). The membrane permeability is represented by the colon cancer cell line (Caco-2)] in our investigated drugs, and the maximum has been observed in ligand 07 (1.058 × 10^−6^). The VDss (human) level varies from −0.495 to 0.277. Another critical parameter is BBB, and these data were collected from SwissADME online tools, which indicate that only ligand 07 can cross the BBB, and the other 10 ligands cannot pass the BBB. Since metabolism is investigated based on the cytochrome P450 enzyme for substrate (CYP1A2 and CYP 2C9), the ADME predicted table revealed that maximum ligand can be metabolized or inhibited in CYP450 1A2 inhibitor and CYP450 1A2 inhibitor 2C9. The pkCSM model has been implemented to measure the pharmacokinetics of the total clearance log (CLtot) of a mentioned compound in the log(ml/min/kg). The higher the CLtot ranges of the ligands, the faster the excretion rate, and it is revealed that ligand 02 has maximum clearance rates (0.492 ml/min/kg). The different biochemical and ADME features of the ligands demonstrated that all candidates have excellent pharmacokinetic properties (**Table 4**).

**Table 4:** Summary of ADME results for 11 selected derivatives of genistein.

Ligand no.	Absorption		Distribution		Metabolism		Excretion	
	Log S*	Coco-2 P* Y (10^−6^ cm/s)	VDss (human)	BBB-P*	CYP450 1A2*	CYP450 2C9*	TC* (ml/min/kg)	R-OCT2*
01	−3.428	1.024	−0.495	No	Yes	No	0.248	No
02	−3.517	0.808	−0.351	No	Yes	No	0.492	No
03	−2.892	0.816	0.011	No	No	No	−32.729	No
04	−2.995	0.760	−0.033	No	Yes	Yes	0.375	No
05	−2.921	0.485	0.017	No	Yes	Yes	0.309	No
06	−3.472	1.024	−0.05	No	Yes	Yes	0.389	No
07	−3.525	1.058	−0.01	Yes	Yes	No	0.423	No
08	−5.035	0.951	−0.731	No	Yes	Yes	0.288	No
09	−2.892	−1.224	0.011	No	Yes	No	−1.936	No
10	−3.763	−0.219	0.077	No	Yes	No	0.434	No
11	−3.732	−0.174	0.277	No	No	Yes	0.471	No

*Water solubility, LogS; Permeability, Caco-2 permeability, VDss, volume of distribution, Blood–brain barrier permeability, BBB-P, Cytochrome 450 1A2 inhibitor, CYP450 1A2, Cytochrome 450 2C9 inhibitor, CYP450 2C9. Total clearance, TC; Renal OCT2 substance, R-OCT2.

### 4.6 Aquatic and non-aquatic toxicity

Numerous drugs or potent molecules fail after development or during development stages due to aquatic and non-aquatic toxicity, which impacts the ecosystem and phycological system. Thus, aquatic and non-aquatic toxicity parameters have a significant rule to be established and made commercially available in the market. AMES toxicity, max. tolerated dose, oral rat acute toxicity, oral rat chronic toxicity, hepatotoxicity, skin sensitization, and *T. pyriformis* toxicity were listed in the table to evaluate the toxicity level. In AMES toxicity level, ligands 01, 02, 04, 06, 07, and 11 have no effects, while the remaining ligands positively respond to AMES toxicity. It signifies that the chemical (03, 05, 08, 09, and 10) is carcinogenic and, as a result, can potentially cause cancer. The tolerated dose (human) is approximately 1.068 mg/kg/day in ligand 11, indicating that if a person should take higher than 1.068 mg/kg/day, this drug should produce toxicity. The oral rat acute toxicity (LD_50_) level (1–2.678 mol/kg) and the oral rat chronic toxicity level (1–50.324 mg/kg/day) indicate that the compounds may be lethal if given at very high doses. No drugs affect skin sensitization while only ligands 07 and 11 may produce hepatotoxicity (**Table 5**).

**Table 5:** Summary of aquatic and non-aquatic toxicity results for 11 selected derivatives of genistein.

S/N	AMES-T*	M.TD* (human) mg/kg/day	ORAT (LD_50_) (mol/kg)	ORCT (mg/kg/day)	HT	SS	T.P Tox (log μg/L)
01	No	0.728	1.907	1.682	No	No	0.524
02	No	0.779	2.374	1.424	No	No	0.336
03	Yes	0.438	2.482	16.892	No	No	0.285
04	No	0.578	2.542	1.877	No	No	0.355
05	Yes	0.472	2.597	1.991	No	No	0.376
06	No	0.646	2.529	1.577	No	No	0.419
07	No	0.638	2.503	1.271	No	No	0.511
08	Yes	0.568	2.678	0.687	Yes	No	0.291
09	Yes	0.438	2.482	50.324	No	No	0.285
10	Yes	0.702	2.362	1.766	No	No	0.334
11	No	1.068	2.276	2.057	Yes	No	0.303

*AMES toxicity, AMES-T; Max. tolerated dose, M.TD; Oral rat acute toxicity, ORAT; Oral rat chronic toxicity, ORCT, Hepatotoxicity, HT, Skin sensitization, SS, T. pyriformis toxicity, T.P Tox.

### 4.7 Molecular dynamics simulations

The goal of MD simulation is to get a real-time understanding of the stabilization and intermolecular connection of a protein–ligand complex. This method may also be used to quantify the structural change that occurs in a complex system after it has been subjected to an enclosed environment (57).

In this study, a 100-ns MD simulation of the protein in contact with a particular ligand was performed to comprehend the protein**’**s conformational changes. The terminal illustration of MD simulations results at 100 ns, and movements of the molecule were stabilized for evaluation of the intermolecular dynamics.

**Figure 4:**
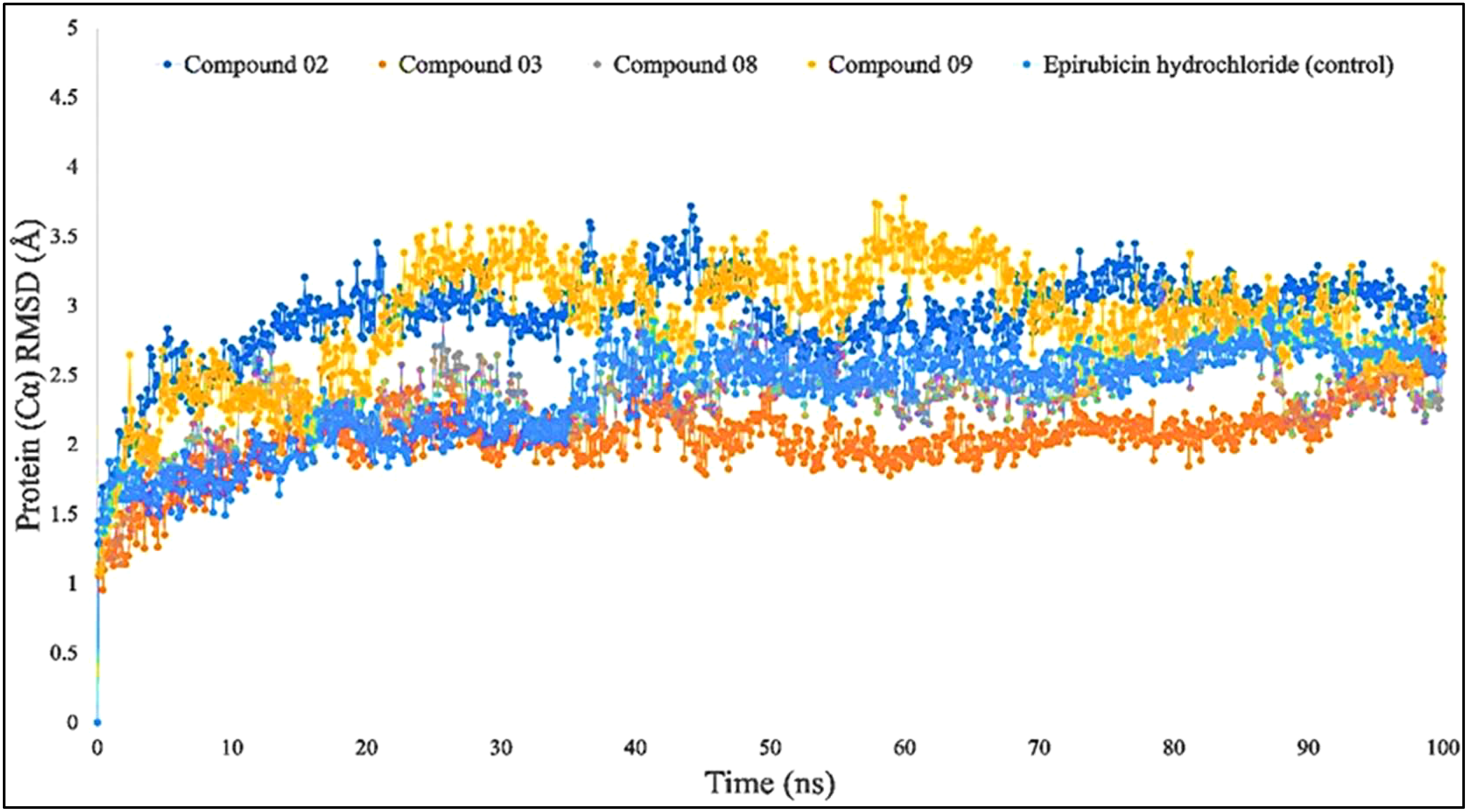
Comparison of RMSD values among the top 5 compounds: 02, 03, 08, 09, and epirubicin hydrochloride.

When the RMSD range of 1–3 Å is present during the simulation, it has been counted that the ligand–protein is bound with each other perfectly, and smaller deviations suggest that the complicated structure is more stable than it seems. This shows that the protein**’**s structure has changed significantly if the RMSD value is larger than 1–3 Å. In light of this investigation, the MD simulations were carried out for a period of 100 ns in order to ascertain the conformational change that occurred in the target protein since it was in the presence of the four ligand substances L02, L03, L08, and L09, as well as epirubicin hydrochloride. The blue color represents L02, orange represents L03, gray represents L08, yellow represents L09, and sky blue represents epirubicin hydrochloride. For the bioactive compound L02, the average RMSD was between 1 and 2 Å; L03, 1–1.8 Å; L08, 1–1.8 Å; L09, 1–3 Å; and epirubicin hydrochloride, 1–2.5 Å. In this investigation, the RMSD score changed with the change of the period, and finally, fell in the 1–3 Å range at 100 ns, which indicates that they are perfectly bonded with the targeted protein (**Figure 4**).

**Figure 5:**
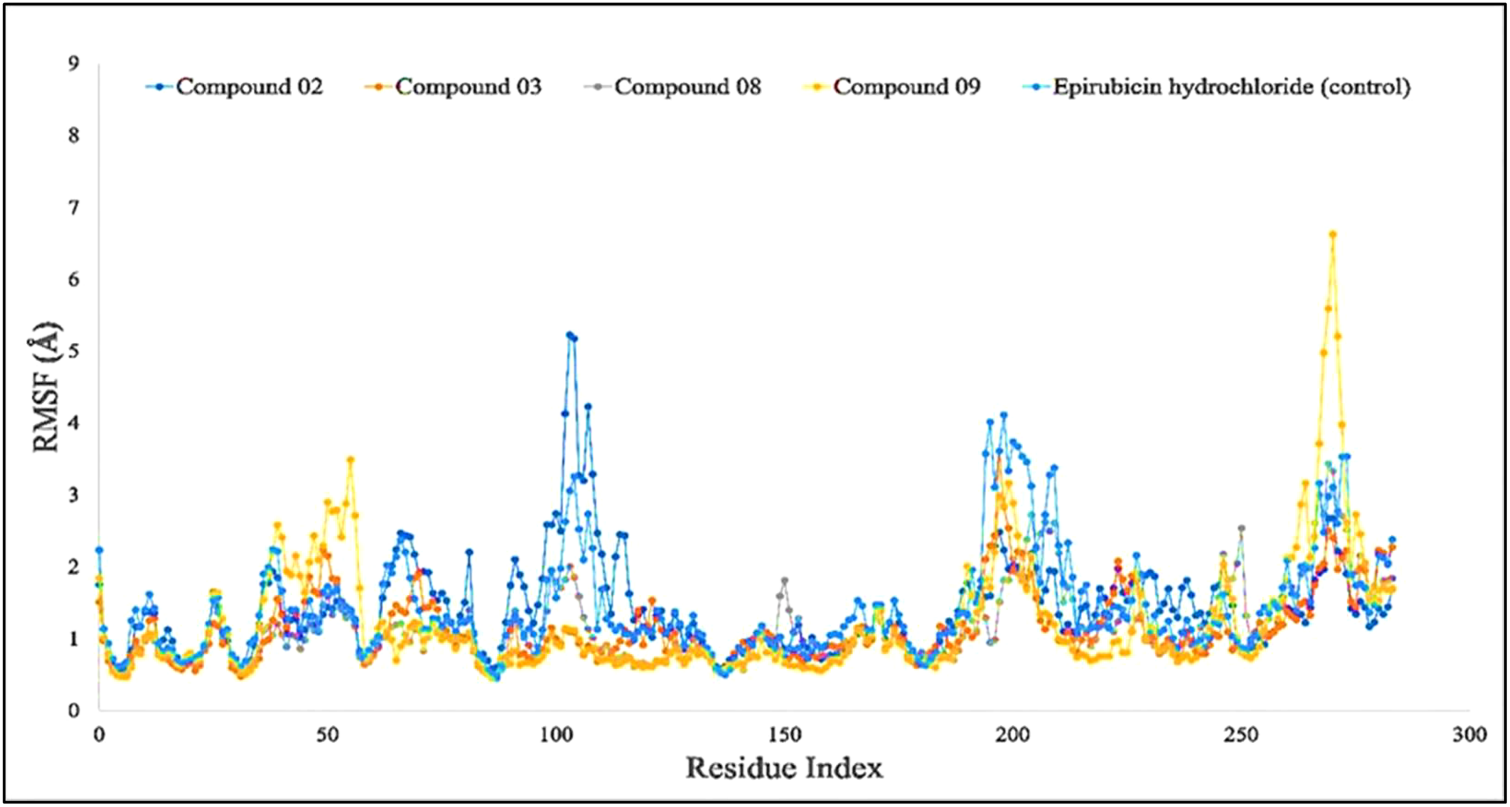
Comparison of RMSF values among the top 5 compounds: 02, 03, 08, 09, and Epirubicin hydrochloride.

The RMSF may help characterize and determine the specific changes occurring in the protein chain when specific ligand molecules bind with distinct positions, and it is useful for obtaining details on the fluctuations that occur per residue in protein–ligand complexes (58). As seen in **Figure 5**, the RMSF values of compounds L02, L03, L08, and L09 in association with the breast cancer protein (PDB ID: 3HB5) were determined to examine the modification in protein structural plasticity induced by the binding of designated ligand molecules to a certain residual location. **Figure 5** represents the RMSF values measured for the complex of breast cancer protein (PDB ID: 3HB5) in the docked protein–ligand complex. Since this study has been performed based on higher binding energy, it is clearly understood that the RMSF score is also much better in a stable configuration.

The average RMSF values are presented at 4.2 Å. Each colored peak presented the RMSF fluctuation values among five docked complexes.

The radius of gyration, often known as Rg, was calculated so that investigators could evaluate how efficient the protein was both in the absence and the presence of simulated impacts, and the distribution of a protein–ligand interaction system**’**s atoms along its axis is one way to describe the properties. The computation of Rg is one of the most critical signs to look for when attempting to forecast the structural functioning of a macromolecule. This is because it exposes variations in the complex efficiency over time, which is among the most important things to determine. The Rg investigation provides a comprehensive view of the bending and expansion of the protein structure as a consequence of the interaction of simulation hits.

**Figure 6:**
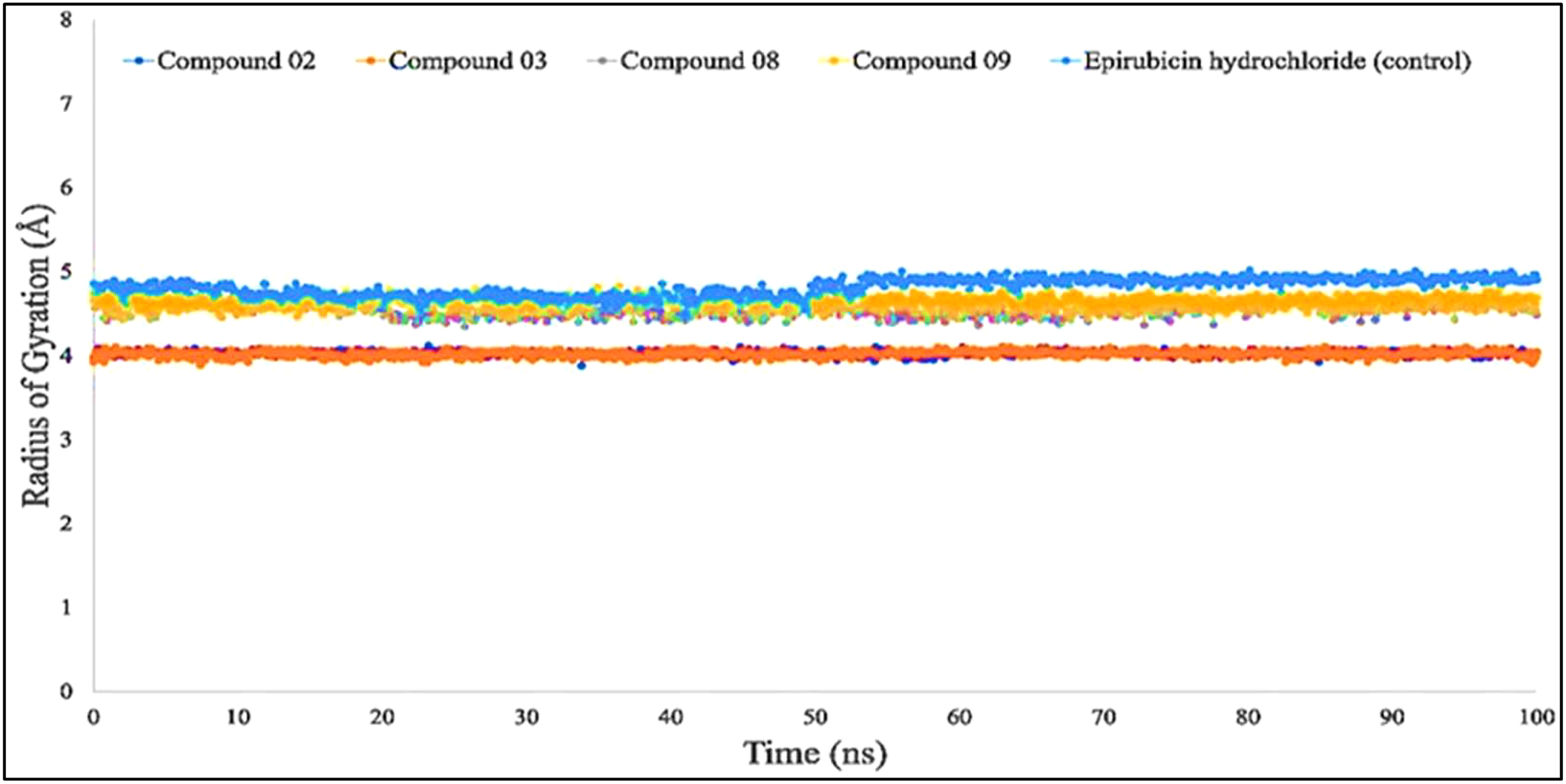
Comparison of the radius of gyration values among the top 5 compound docked complexes: 02, 03, 08, 09, and epirubicin hydrochloride.

According to the finding in **Figure 6**, the average Rg values have been computed for breast cancer protein (PDB ID: 3HB5); L02, L08, L09, and standard epirubicin hydrochloride obtained a value of 4.5–4.70, while L03 obtained an Rg score of 4.0 from the beginning to the endpoint. According to the findings, the binding of compounds L02, L03, L08, and L09 resulted in a considerable enhancement of the stability.

Protein or biological macromolecule folding and stability investigation have historically relied heavily on the amount of solvent-accessible surface area (SASA) of the protein. It is described as the surface that is circumscribed around a protein by a theoretical center of a solvent spherical that is in touch with the van der Waals contact surface of the biological macromolecules. It has been regarded as amino acid residues on a protein**’**s surface that act as potential binding sites and/or engagement with the potential drug candidate. This feature enhances the characteristics of biological macromolecules including hydrophilicity or hydrophobicity and also protein–ligand binding relation.

**Figure 7:**
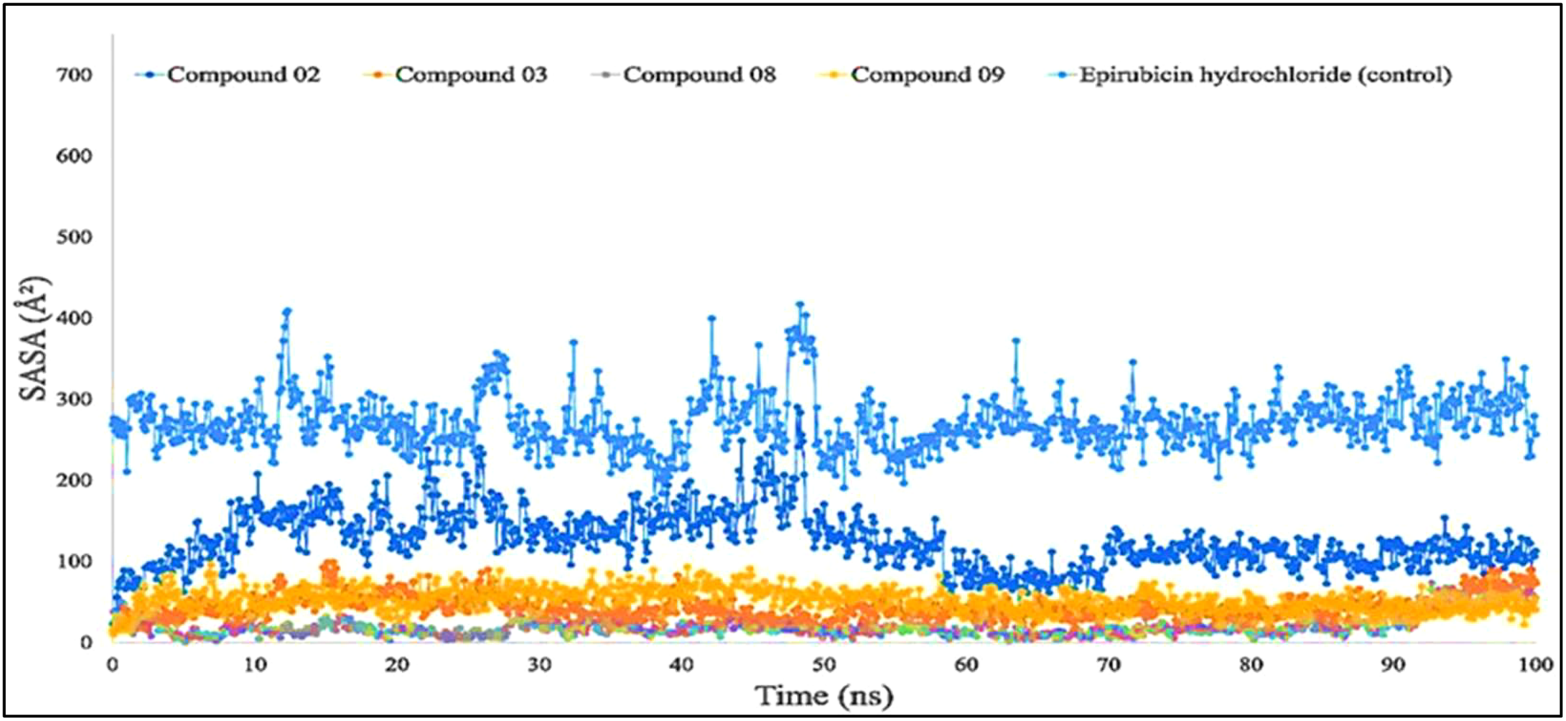
Comparison of solvent accessible surface area (SASA) values among the top 5 compounds: 02, 03, 08, 09, and epirubicin hydrochloride.

**Figure 7** shows that protein–ligand engagement was utilized to derive the SASA of the substances involved in the interaction. The reported bioactive molecules L02, L03, L08, L09, and standard epirubicin hydrochloride have been displayed in blue, sky blue, orange, yellow, and gray, respectively. The bioactive molecules had an average SASA value of 0 to 410 A**^2^**, suggesting that a high proportion of the designated ligand molecules was present in a complicated medium.

**Figure 8:**
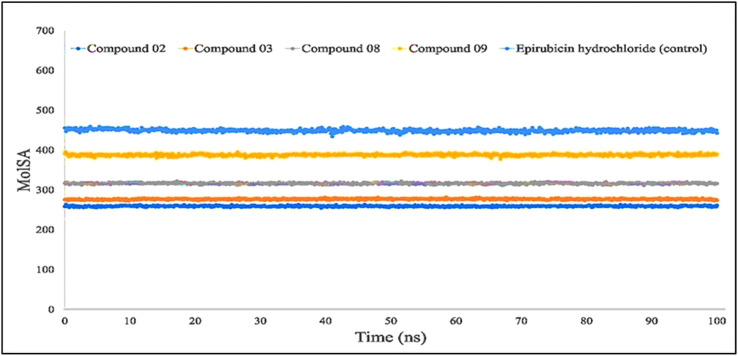
Comparison of solvent molecular surface area (MolSA) values among the top 5 compounds: 02, 03, 08, 09, and epirubicin hydrochloride.

The molecular surface area, also known as the MolSA, is comparable to the van der Waals surface area, and it has been computed using a probe radius of 1.4 Å. In the investigation of the current research, ligands L02, L08, and L09 have obtained a better van der Waals surface area (**Figure 8**).

**Figure 9:**
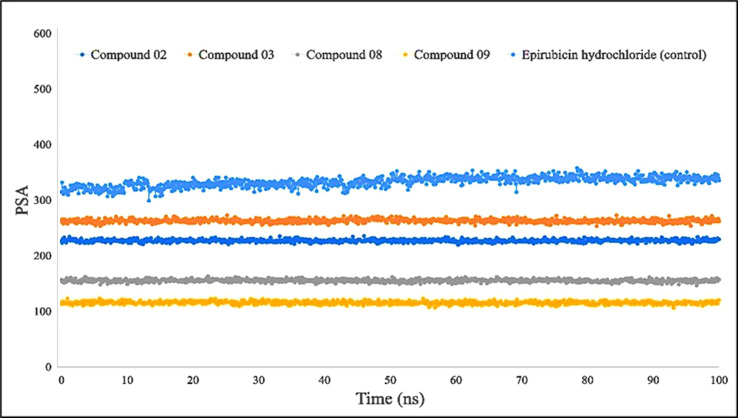
Comparison of polar surface area (PSA) values among the top 5 compounds: 02, 03, 08, 09, and epirubicin hydrochloride.

Furthermore, primarily oxygen and nitrogen atoms participate in a substance**’**s PSA while all three ligands (L02, L08, and L09) had high PSA values when tested with the specific biological macromolecules or proteins (**Figure 9**). PSA has been demonstrated to help with cellular efficiency, intestinal permeability, and BBB penetration or limitation to peripheral circulation (59). The protein–ligand engagement schematic was used to derive the calculations to determine the required PSA of the bioactive reported molecules involved in the formation of the protein–ligand complex. The finding of this investigation has been reported from 100 Å to 310 Å, which could be described as outstanding to be a potent medication.

**Figure 10:**
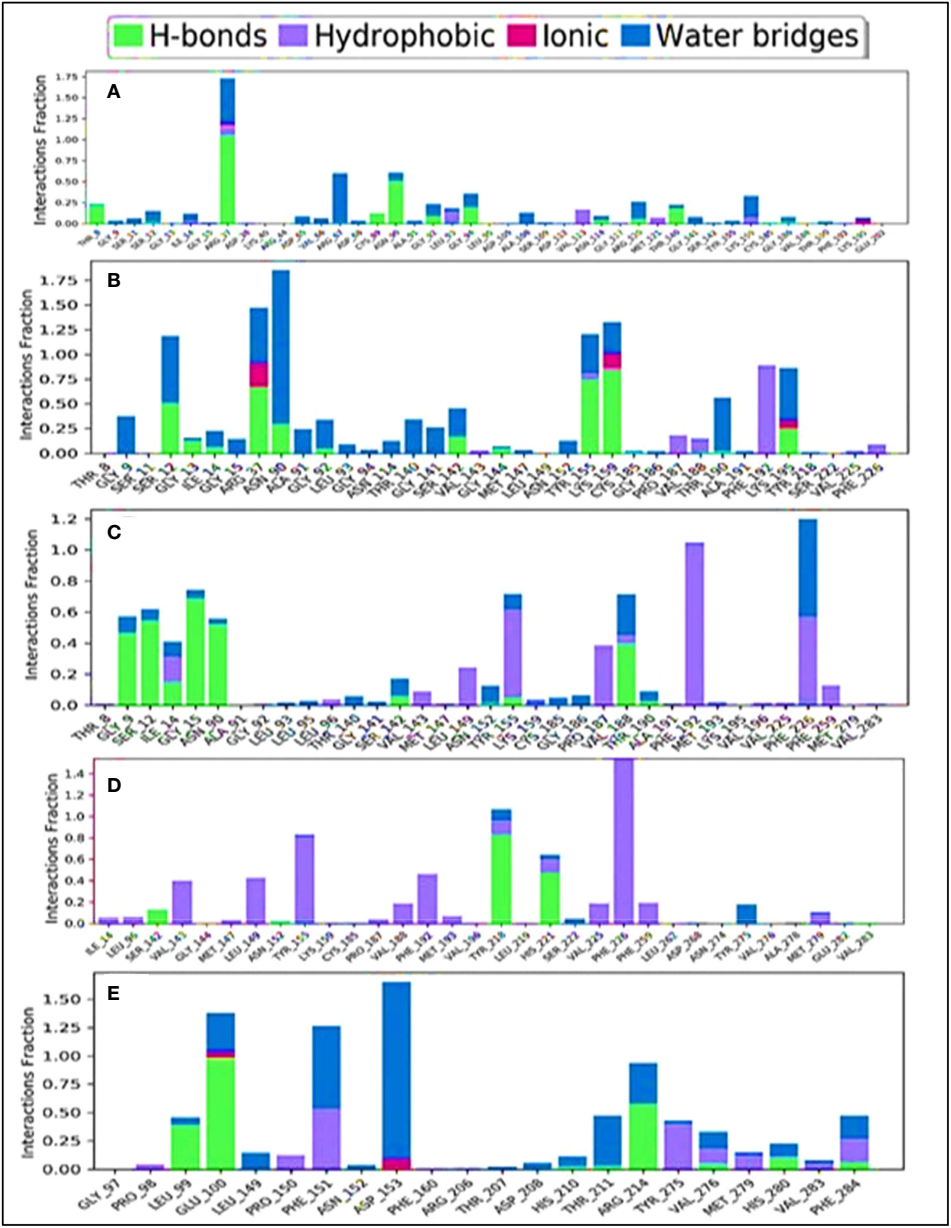
Comparison of intramolecular bond analysis values among the top 5 compounds: 02 **(A)**, 03 **(B)**, 08 **(C)**, 09 **(D)**, and epirubicin hydrochloride **(E)**. Each colored bar presents a specific type of binding interaction among the docked complexes of the five compounds.

The combination of the complex structure of a protein with the ligands consisted of different intermolecular interactions during the simulation of 100 ns using a tool called the simulation interactions diagram (SID) (60). The dependencies between macromolecular proteins and bioactive ligands that were found during the 100-ns simulation are displayed as stacked bar charts in **Figure 10**. The four bioactive molecules and standard epirubicin hydrochloride have been analyzed and graphically represented in this portion, such as L02, L03 L08, and L09 and standard epirubicin hydrochloride engaging in or binding with selected breast cancer macromolecules. Several different bonds have been seen during the formation of the protein–ligand complex including the non-covalent bond (hydrophobic bond), the hydrogen bond, the ionic bond, and the water bridge bond. During the 100-ns simulation, it is revealed that all molecules developed and produced several molecular couplings with the particular protein involving hydrogen, hydrophobic, ionic, and water bridge bonds, which finally help them form a stable configuration.

The engagement schematic (**Figure 10**) from the 100-ns simulation was used to compute the molecular surface area, abbreviated as **“**MolSA,**”** of the protein–ligand engagement complexes. The colors blue, orange, gray, and yellow were used to represent the documented bioactive compounds L02, L03, L08, and L09, respectively. The standard epirubicin hydrochloride is also included, shown in sky blue.

Through the use of MD modeling, it should be verified that a protein can maintain its stability while bound to ligands and can form a stable configuration, which also verifies the molecular docking simulation (61). It has also been able to obtain tightness, which described protein–ligand complexes remaining stable in a certain environment after reaching a psychological system. The maximal consistency of a biomolecule is reflected by its RMSD, while the mean fluctuations that characterize the tightness of the protein–ligand complex are defined by its RSMF measurements (62). The protein–ligand complex**’**s activities were used to compute the system**’**s RMSD, which confirmed the protein**’**s low degree of variability or minimum fluctuation. The RMSF measurement was utilized to determine the fluctuation of the protein, which indicated a decreased fluctuation overall, thus confirming the compound**’**s consistency regarding the target protein. According to the finding, the selected breast cancer protein and all four of its ligands (L02, L03, L08, and L09) have shown significant RMSD and RMSF values.

For Rg, the center of mass from the C and N terminals of the protein has been estimated, which investigated the protein reinforcement and offered a more comprehensive evaluation of protein folding properties (63). The elevated Rg quantity, on the other hand, implies that the chemicals have been disassociated from the protein, while the lower the Rg score, the greater the tightness. The structure has been considered less stable when the SASA value is larger, and the complex of water molecules and amino acid residues is more densely compacted when the SASA value is greater. According to our findings, all of the ligand-tested compounds had optimal Rg, SASA, MolSA, and PSA scores to be potent drug candidates.

## 5 Conclusion

Breast and lung cancer are major global health concerns, prompting the need for effective treatments. Although targeted therapy has progressed, side effects and acquired resistance remain challenges. Existing FDA-approved drugs and chemotherapies are costly and often ineffective. Thus, novel agents like genistein, derived from *Glycyrrhiza glabra* (licorice), offer hope in combating breast and lung cancers, according to our *in silico* findings. In our present CADD study, we designed 11 genistein derivatives through modification of side chains and functional groups. This investigation revealed significant protein–ligand docking interactions, stable conformations during MD simulations, favorable drug-like properties, and reliable PASS prediction for all derivatives. As a result, these compounds demonstrate promise as bioavailable oral drugs for breast and lung cancer treatment.

Notably, compounds 08 and 09 showed particularly strong binding affinities of −11.0 kcal/mol and −10.0 kcal/mol against breast cancer (PDB ID: 3HB5). Ligands 03 and 09 also displayed notable binding energies of −8.8 kcal/mol and −9.5 kcal/mol against lung cancer, with minimal concerns regarding AMES or hepatotoxicity. The majority of the drugs met ADMET profile parameters, exhibiting good water solubility and high gastrointestinal absorption. Additionally, drug 07 showed potential in crossing the BBB.

Further validation through 100-ns MD simulations of selected complexes (L02, L03, L08, and L09) confirmed their stability in suppressing targeted proteins, with minor fluctuations in RMSD and RMSF measurements. Moreover, these ligands demonstrated promising outcomes in SASA, Rg, MolSA, and PSA evaluations. Finally, it should be summarized that these findings suggest that the designed compounds hold great promise as alternative and improved treatment options for breast and lung cancer, and further experimental work should be carried out.

## Data availability statement

The raw data supporting the conclusions of this article will be made available by the authors, without undue reservation.

## Author contributions

Conceptualization: SA and SB; methodology: SA, PB, and NS; software and visualizations: SA, SB, PB, DK, MNH, and MEH.; validation: SB, SA, PB, and H-AN; data curation: SA, PB, DK, MB, and YJ; writing—original draft preparation: SA, SB, PB; writing—review and editing: H-AN, YJ, and NS; investigation and supervision: SB and MB. All authors have read and agreed to the published version of the manuscript.
